# The Effects of Environmental and Management Factors on the Quality of the Corpus Luteum and Subsequent Conception Rates of Beef and Dairy Cows in South Africa

**DOI:** 10.3390/life15111687

**Published:** 2025-10-30

**Authors:** Kaylee Demont, Edward Cottington Webb, Robert Treadwell, Andries Masenge

**Affiliations:** 1 Department of Animal Science, Production Animal Physiology Research Group, Faculty of Natural and Agricultural Sciences, University of Pretoria, Pretoria 0028, South Africa; u16033923@tuks.co.za; 2 Department of Animal Science, Tarleton State University, Texas A&M University System, Stephenville, TX 76401, USA; 3 Embrio Plus, 41 Hendrik Verwoerd Ave, Brits 0250, South Africa; robert@embryoplus.co.za; 4 Department of Statistics, Faculty of Natural and Agricultural Sciences, University of Pretoria, Pretoria 0028, South Africa; andries.masenge@up.ac.za

**Keywords:** reproduction, beef, dairy, conception rate, climatological effects, management, embryo transfers, corpus luteum

## Abstract

The corpus luteum (CL) controls the success of pregnancy. The current study investigated the effects of some environmental and management factors on the development of the CL and the subsequent impact on conception rates of beef and dairy cows in South Africa. Cows (Jersey, Holstein, Nguni, Bonsmara, Tuli, Ankole, Charolais and Angus crosses) receiving an embryo were synchronized based on the breed type and status. Standing estrus was observed from day 9 to day 12 after synchronization. Embryo transfer occurred on day 18. CLs were graded based on size and consistency of each CL by an experienced veterinarian. Production type (beef versus dairy types) was marginally associated with conception after the first synchronization cycle (*p* = 0.065). Cows were moved between groups, and this influenced the CL (*p* = 0.08). Climatological factors, maximum THI (*p* = 0.017) and precipitation (*p* = 0.061) influenced the quality of the CL in dairy cows. By contrast, precipitation (*p* = 0.067) influenced the quality of CLs in beef cows. More attention needs to be paid to the management of maximum THI and shelter from precipitation in dairy production systems, while precipitation alone was more important in beef cows. Climate affects the corpus luteum quality of cows of different production types differently.

## 1. Introduction

The effect of the size of the corpus luteum on conception rate has been considered previously in a Nellore and Angus cross [1], Herefords [2] and Holstein Friesian cattle [3,4,5]. These studies considered the physiological effects of CL size on conception rates in different breeds and types of cows. Progesterone production is vital for the maintenance of pregnancy, especially in early gestation [6]. The size of the CL differs between *Bos indicus* (17 to 21 mm) and *Bos taurus* (20 to 30 mm) cows [1,3,4,7]. Furthermore, the quality of the CL is influenced by the growth and development of the CL, which influences conception rate. There is a finite space for agricultural land, and we are fast running out of space. There is a need for more efficient ways to supply meat to meet the world’s growing population needs. [8]. Environmental factors are diverse, and their effect on the quality of the corpus luteum will be dependent on the adaptability of the species [8]. Selection due to management practices has resulted in an increased growth rate but is associated with a direct negative correlation with reproductive efficiency. Looking at the effects of environmental factors on corpus luteum quality can contribute to the maintenance of reproductive efficiency in the selection process.

Both beef and dairy production are vital in South Africa [8]. The South African semi-arid climate can be unforgiving to animals that are not adapted to it. Long, hot summer and cool, dry winter are prominent seasons, while spring and autumn are more moderate. Species adapted to this are less affected reproductively. Weather factors can affect the mature weight of a cow, which has been shown to influence adaptability [9,10]. Specific weather factors such as THI are important because they consider the interaction of weather factors and not standalone factors, which is important for adaptation. The aim of the study was to determine the effect of some environmental and management factors that influence the development of the CL and the subsequent effects on the conception rates of beef and dairy cows in the North West province of South Africa. The hypothesis states that the quality of the corpus luteum will be affected by the various environmental factors in South Africa and that the severity is dependent on the type of production of the cow.

## 2. Materials and Methods

Ethical approval was obtained for the use of data from the Ethics Committee of the Faculty of Natural and Agricultural Sciences at the University of Pretoria (NAS359/2022). Cows were kept at Embrio Plus farm in Brits, North West province (−25.64091023156352, 27.78773330910706; 1105 m altitude). All cows that were at EmbryoPlus during the duration were included in the study (September 2021 to January 2023).

Breeds included Jersey, Holstein, Nguni, Bonsmara, Tuli, Ankole, Charolais and Angus crosses. There were approximately 500 cows that could be considered during that period. The numbers are not evenly distributed between breed types, but this can be accounted for in statistical analysis through the mixed model. Heifers were cows that had not reached mature weight and size.

Cows brought into the facilities were given a backgrounding period to allow for adaptation in a camp (25 m × 100 m). All cows were fed the same diet (ad libitum weeping love grass (*Eragrostis curvula*) + Wildswinkel Recipient concentrate (between 2–4 kg/animal/day, depending on lactation status/condition/breed/age)). Cows in cycle 1 conceived on their first synchronization program, cows in cycle two conceived in their second synchronization program, and cows in cycle three conceived on their third synchronization program. In each cycle (cycle 1 = n1; cycle 2 = n2; cycle 3 = n3), the cows ready for embryo flushing were included (n1 = 525; n2 = 297; n3 = 150). This study included data from cows enrolled from September 2021 to January 2023. All cows were kept in similar camps of 25 m × 100 m in size.

### 2.1. Synchronization Protocol

The treatment procedures involved restraining cows in a crush, followed by pregnancy checks and synchronization based on the breed type (Table 1). All cows received a trace-mineral injection of Multimin^®^ (+Cu, Zn, Se) from Virbac South Africa (Centurion, South Africa) subcutaneously and 100 mL oral supplementation of Atlantic Gold^®^ vitamin A & D (Reg No. V23068, Act 36 of 1974, Biorem Biological Products, Oudtshoorn, South Africa) as early as possible before cows were fitted with an intra-vaginal progesterone-releasing device (CIDR^®^B; 1.9 g of P4 from Zoetis South Africa, Sandton, South Africa) on day 0 and injected with 2 mL of estradiol benzoate (compounded by V-tech, Hong Kong) intramuscularly.

**Table 1:** Table showing the total number of cycles considered for each breed for the duration of the study.

Breed	Total Cycles	Breed	Total Cycles
Ankole	20	Jersey	454
Ankole cross	83	Jersey cross	19
Brahman cross	59	Wagu cross	18
Bonsmara	121	Nguni	242
Bonsmara cross	7	Nguni cross	2
Boran double cross	10	Charolias	1
Boran cross	14	Charolias cross	2
Chianinia	4	DB	1
Chianina Jersey cross	3	Simbra	1
Holstein	29	Simentaler	1
Holstein cross	11	Tuli	1

On day 8, the CIDR was removed, and each cow was treated with intramuscular injections of 3.3 mL of Chronogest^®^ (PMSG 6000 IU; MSD Animal Health, South Africa, Kempton Park, South Africa), 1 mL of Oestradiol cypionate^®^ (sodium cloprostenol; compounded by V-Tech) and 2 mL of Estrumate^®^ (1 mL of i/m cloprostenol from MSD Animal Health, South Africa). Standing estrus observations were performed in the morning and the evening for the following three days (days 9 to 12), and embryo transfer occurred on day 18. Jersey heifers were given a lower dosage of PMSG, and possibly pregnant females were only given CIDR (CIDR^®^B; 1.9 g of P4 from Zoetis South Africa) devices and no injections.

Possibly pregnant cows are kept in synchronization programs. Pregnancy diagnosis is performed early, at 6 weeks. It can be difficult to determine the pregnancy at the time. If there is a chance that the cow is pregnant, it is placed into the “Possibly pregnant” program. This allows the cow to stay synchronized if it is not found to be pregnant at the 9-week check.

If a cow shows signs of estrus within the first 4 weeks after embryo transfer, it is examined 7 days after that display. This allows the veterinarian to determine the position of the CL. If the CL is on the same side as the embryo transfer had happened 4 weeks previously, the cow is left for the subsequent 2 weeks so that a pregnancy diagnosis can be performed. If the CL is on the opposite side of the previous embryo transfer, then the recipient receives another embryo that day. If there is no CL present, this is where the cow is placed in the possibly pregnant group. It is treated with a CIRD only, as other treatments could harm a possible pregnancy.

Table 2 shows how many cycles were considered for each type of synchronization program in different seasons.

**Table 2:** Number of animals assigned to each synchronization program in cycle 1, 2, or 3 of their programs.

Summer	Autumn	Winter	Spring
*Bos indicus* synchronization Cycle 1 n = 90 Cycle 2 n = 27 Cycle 3 n = 5	*Bos indicus* synchronization Cycle 1 n = 25 Cycle 2 n = 30 Cycle 3 n = 24	*Bos indicus* synchronizations Cycle 1 n = 53 Cycle 2 n = 15 Cycle 3 n = 6	*Bos indicus* synchronization Cycle 1 n = 77 Cycle 2 n = 15 Cycle 3 n = 6
*Bos taurus* synchronization Cycle 1 n = 5 Cycle 2 n = 5 Cycle 3 n = 5	*Bos taurus* synchronization Cycle 1 n = 60 Cycle 2 n = 4 Cycle 3 n = 2	*Bos taurus* synchronization Cycle 1 n = 82 Cycle 2 n = 13 Cycle 3 n = 6	*Bos taurus* synchronization Cycle 1 n = 49 Cycle 2 n = 16 Cycle 3 n = 12
Jersey heifer synchronization Cycle 1 n = 0 Cycle 2 n = 4 Cycle 3 n = 3	Jersey heifer synchronization Cycle 1 n = 4 Cycle 2 n = 0 Cycle 3 n = 2	Jersey heifer synchronization Cycle 1 n = 50 Cycle 2 n = 0 Cycle 3 n = 0	Jersey heifer synchronization Cycle 1 n = 7 Cycle 2 n = 14 Cycle 3 n = 6
Program without injection synchronization Cycle 1 n = 0 Cycle 2 n = 1 Cycle 3 n = 1	Program without injection synchronization Cycle 1 n = 4 Cycle 2 n = 4 Cycle 3 n = 4	Program without injection synchronization Cycle 1 n = 0 Cycle 2 n = 12 Cycle 3 n = 3	Program without injection synchronization Cycle 1 n = 1 Cycle 2 n = 11 Cycle 3 n = 4
No program Cycle 1 n = 2 Cycle 2 n = 2 Cycle 3 n = 7	No program Cycle 1 n = 5 Cycle 2 n = 18 Cycle 3 n = 11	No program Cycle 1 n = 2 Cycle 2 n = 42 Cycle 3 n = 17	No program Cycle 1 n = 10 Cycle 2 n = 34 Cycle 3 n = 34

**Figure 1:**
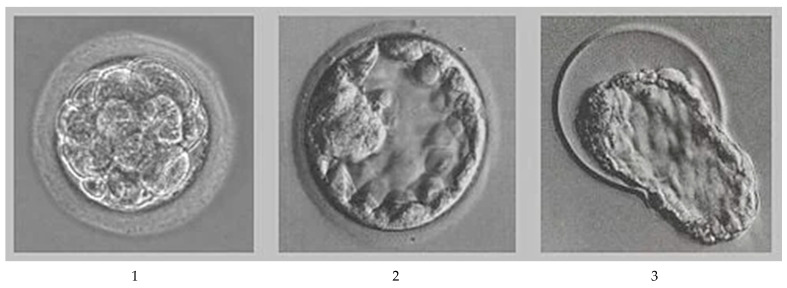
Stages of embryos considered when determining the quality of the embryo (1—morula; 2—blastocyst; 3—hatched blastocyst).

All embryos were graded in the laboratory under a microscope on the day of flushing. Embryos were then either placed in a straw and transferred to the recipient cow or frozen for future use by employing proper storage techniques [6]. Recipient cows were examined via rectal palpation on the day of embryo transfer by an experienced veterinarian. The anatomical side of ovulation, CL grade (described below) and the number of days after standing estrus were determined to assist with selecting the type of embryo for transfer. The embryos are further explained in Figure 1. Each CL was graded via rectal palpation based on size and consistency. The determination of CL size depends on the animal’s age (heifers generally have a smaller CL) and the number of days after standing estrus. The quality of the CLs was determined as outlined in Table 3. The veterinarian determined quality, with the rating being two numbers (1, 2), where 1 would be the grade and 2 would be the day. When the embryo was transferred, this score was used to determine which cow should receive the embryo. The suitability of recipient cows for embryo transfer was determined by clinical examination by the veterinarian. This method was used by the veterinarian, as it is the standard practice of the facility. The facility has been using it for several years, and its use is standardized among the staff.

### 2.2. Embryo Transfer Procedure

The quality of the embryo was determined by the stage that it was in: morula, blastocyst, hatched blastocyst or extended blastocyst (shown in Figure 1).

The quality of the embryo was determined to be morula, blastocyst or hatched blastocyst. Cows were brought into the crush and neck clamped. A sedative injection was given intramuscularly, and a local anesthetic was given as an epidural injection. Rectal palpation was performed to check for a corpus luteum, and the embryo was transferred transvaginally using an embryo pistolette, sheath and sanitary sleeve, in the middle third of the uterine horn.

Group changes were monitored, and the number of times a cow changed groups was recorded. Climatological data was obtained from the nearest South African Weather Services station at Brits in the North West Province (Table 4). The climatological data added was linked to the embryo transfer date.

Summer was from December to February, Autumn was from March to May, winter was from June to August, and Spring was from September to November. These climatological seasons are linked with direct weather factors, which can result in some confounding. THI was calculated using the following equation:


THI=0.8×T+RH×(T−14.4)+46.4

The number of precipitation readings varied due to the n value only containing days where precipitation occurred, showing skewed results for precipitation. While days when no precipitation occurred were still included, the numbers here show the precipitation that did occur.

### 2.3. Statistical Analyses

Statistical analysis was performed using SPSS version 29 by IBM (Armonk, NY, USA) and the SAS version 13 statistical program. Chi-square analysis of pregnancy diagnosis data was used to test the significance of the factors on conception rates. Pearson Chi-square values were considered if the expected count was greater than five, and the Fisher’s Exact test value was considered if the expected count was less than five.

Generalized linear mixed model analyses were used to test the effects of the weather factors on corpora lutea characteristics. Beef and dairy cows were separated for analysis (Table 5). This was based on their main production use as a breed overall.

Animals that had missing values were excluded from specific analyses. Factors that were considered were days after ovulation (day), the size of the corpus luteum (grade) and the side that the corpus luteum was found on (side). The following were also considered: climatological season (summer, spring, winter and autumn), synchronization program (*Bos indicus*, *Bos taurus*, possibly pregnant and Jersey heifer synchronization), breed type (*Bos indicus*, *Bos taurus* or hybrid) and production type (beef production or dairy production).

Significance levels were tested at *p* ≤ 0.05, and tendencies were *p*-values that fell between 0.10 and 0.05. Many of the results fell within those tendencies, showing a tendency toward but not a definitive outcome for those factors.

## 3. Results

The conception rates recorded for embryo recipient cows in the study are presented in Table 6. Approximately 7% of the cows could not be diagnosed confidently by the end of the experimental period. These cows were in synchronization programs, and the CL was measured, but the conception could not be confirmed. The numerical value was the exact number of cycles that resulted in pregnant cows (in calf) and nonpregnant cows (not in calf), and the percentage represents the proportion of the total.

The effects of synchronization cycle, anatomical side of ovulation, embryo grade and day of embryo transfer on conception rates of recipient cows are presented in Table 7.

The current study showed that regardless of the synchronization cycle, the number of days after standing estrus had no significant effect on the conception rate of cows (Table 8). This disagrees with a previous study [11], where embryo transfers on day 8 had higher conception rates than embryo transfers on day 7 [11].

**Table 8:** The effects of the climatological season, synchronization program, production and breed type on the grade of the corpus luteum.

Cycle	Factor	n	Test	Value	df	Asymptotic Sig. (2-Sided)
1	Climatological season ^1^	514	Pearson Chi-square	5.04	3	0.17
	Synchronization program	489	Pearson Chi-square	1.78	2	0.41
	Breed type	483	Pearson Chi-square	3.87	3	0.28
	Production type ^2^	498	Pearson Chi-square	3.42	1	0.07
2	Climatological season ^1^	280	Pearson Chi-square	4.11	3	0.25
	Synchronization program	161	Fisher–Freeman–Halton Exact test	3.77		
	Breed type	269	Fisher–Freeman–Halton Exact test	2.69		
	Production type ^2^	273	Pearson Chi-square	0.03	1	0.89
3	Climatological season ^1^	139	Pearson Chi-square	7.369	3	0.06
	Synchronization program	77	Fisher–Freeman–Halton Exact test	5.076		
	Breed type	138	Fisher–Freeman–Halton Exact test			
	Production type ^2^	139	Pearson Chi-square	2.667	1	0.10

^1^ Climatological season: summer was from December to February, autumn was from March to May, winter was from June to August, spring was from September to November. ^2^ This was based on their main production use as a breed overall.

The production type of the cow tended to influence (*p* = 0.07) the quality of CLs of cows in the first synchronization cycle. Although no effects were observed in cows synchronized in the second cycle, climatological season had a strong tendency (*p* = 0.06), and the production type showed a tendency to affect (*p* = 0.10) CL quality in the subsequent cycle.

The mixed model analysis of the effects of different climatological factors on CL quality is shown in Table 9. This analysis showed that the maximum THI influenced CL quality (*p* = 0.017), while daily precipitation (*p* = 0.061) and change in temperature (*p* = 0.1) were marginally associated with CL quality (Table 9) within various breed types. In the beef cows, daily precipitation tended to influence (*p* = 0.067) the quality of the CLs. By contrast, in dairy cows, maximum THI influenced (*p* = 0.017) the quality of the CLs, and both daily precipitation (*p* = 0.061) and the change in daily temperature tended to influence the CL quality.

The change in temperature interacted with the average daily humidity ( $p_{dairy}$ = 0.033, $p_{beef}$ = 0.021) in beef and dairy cows. Average temperature interacted with average humidity in beef cows only ( $p_{beef}=0.004)$, and the maximum THI interacted with wind speed ( $p_{beef}=0.037)$.

As the factors that showed tendencies in both beef and dairy cows, average temperature interacted with daily precipitation ( $p_{dairy}$ = 0.067; $p_{beef}=0.083$), maximum THI ( $p_{dairy}$ = 0.099) and humidity ( $p_{dairy}$ = 0.059). Average wind speed interacting with daily humidity also showed a tendency in beef cows ( $p_{beef}=0.097)$.

**Table 9:** The effects of climatological factors and their interactions on the grade of the corpus luteum in beef- and dairy-type cows.

Fixed Effects	Beef			
Source	F	Df1	Df2	Sig.
Corrected model	14.342	67	313	0.000
Average temperature	1.201	3	313	0.309
Change in temperature	0.298	3	313	0.827
Daily precipitation	2.412	3	313	0.067 *
Average daily humidity	1.904	3	313	0.129
Maximum THI	1.901	3	313	0.129
Minimum THI	0.071	3	313	0.975
Average wind speed	1.167	3	313	0.322
Average temperature × daily rain	2.406	3	313	0.067 *
Average temperature × average daily humidity	2.508	3	313	0.059
Average temperature × average wind speed	1.298	3	313	0.275
Change in temperature × daily precipitation	1.343	3	313	0.260
Change in temperature × average daily humidity	2.957	3	313	0.033 **
Change in temperature × average wind speed	0.080	3	313	0.971
Daily precipitation × average daily humidity	0.318	3	313	0.813
Daily precipitation × maximum THI	2.109	3	313	0.099 *
Daily precipitation × minimum THI	1.928	3	313	0.125
Daily precipitation × average wind speed	1.048	3	313	0.371
Average daily humidity × average wind speed	0.359	3	313	0.782
Maximum THI × average wind speed	1.269	3	313	0.285
Minimum THI × average wind speed	0.725	3	313	0.538
**Fixed Effects**	**Dairy**			
Source	F	Df1	Df2	Sig.
Corrected model	0.604	41	285	0.974
Average temperature	1.288	2	285	0.277
Change in temperature	2.318	2	285	0.100
Daily precipitation	2.823	2	285	0.061 *
Average daily humidity	1.338	2	285	0.264
Maximum THI	4.130	2	285	0.017 **
Minimum THI	0.343	2	285	0.710
Average wind speed	1.125	2	285	0.326
Average temperature × daily rain	2.516	2	285	0.083 *
Average temperature × average daily humidity	5.729	2	285	0.004 **
Average temperature × average wind speed	1.782	2	285	0.170
Change in temperature × daily precipitation	0.824	2	285	0.440
Change in temperature × average daily humidity	3.913	2	285	0.021 **
Change in temperature × average wind speed	0.849	2	285	0.429
Daily precipitation × average daily humidity	0.076	2	285	0.927
Daily precipitation × maximum THI	1.206	2	285	0.301
Daily precipitation × minimum THI	2.075	2	285	0.127
Daily precipitation × average wind speed	0.742	2	285	0.477
Average daily humidity × average wind speed	2.350	2	285	0.097 *
Maximum THI × average wind speed	3.327	2	285	0.037 **
Minimum THI × average wind speed	0.011	2	285	0.989

* Tendency, 0.05 < *p* < 0.1. ** Significant, *p* < 0.05.

## 4. Discussion

The primary objective was to consider the environmental and managerial factors that influenced the quality of the corpus luteum. The generalized linear mixed model of physiological factors revealed that no physiological factors significantly influenced the conception rate. This was an important initial observation due to the need to compare factors that could affect quality. Studies have shown that cows are more likely to ovulate from the right ovary [12], as more follicles are present on the right ovary [12,13]. The first ovulation after pregnancy is most likely to happen in the opposite ovary to the one that the pregnancy was on [14]. The anatomical side of ovulation did not affect the conception rate of recipient cows. It was previously shown that the ovary containing more follicles generally also contains the dominant follicle [12]. In the present study, more ovulations occurred on the right ovary, but this did not influence the conception rates of cows in any of the synchronization cycles (Table 7).

Most commonly, embryo transfer occurs at about day 7, when the embryo is in the blastocyst stage [15,16] and maternal recognition will occur [16]. Previous research [17] indicates that progesterone concentrations of 2–2.5 ng/mL the day before or the day of embryo transfer may result in higher pregnancy rates [17]. It was also reported that cows with a shorter luteal phase had a negative effect on conception [15]. The present study showed that there were no significant effects of corpus luteum quality or the immediate number of days after ovulation (8, 7, or 6 days). The methods used for this study mitigated the inconsistency that can sometimes occur. Due to the cows being so thoroughly examined for suitability in the embryo flushing program, as well as the consideration of matching the age of the embryo to the number of days after estrus detection, variability has been removed compared to the study presented in [11], where similar-age embryos were given on different days.

The CL’s ability to produce sufficient progesterone is reliant on the number of granulosa cells in the ovulatory follicle. Ref. [18] reported that the diameter of the follicle had a quadratic response to the conception rate in *Bos taurus* beef females. This relationship was not detected in *Bos indicus* beef females [18,19]. Corpus luteum diameter and blood flow were moderately correlated in beef cattle [20]. The present study did not have any significance related to corpus luteum grade, irrespective of the breed, but it was unable to link the quality of the corpus luteum to the progesterone concentration. The number of granulosa cells produced could be linked to the size of the CL and therefore the progesterone concentration, but this could not be verified in this study.

The observation that dairy and beef production types of cows influenced the quality of CLs may be linked to adaptation. In order to answer the hypothesis, the environmental factors were each considered as individual fixed factors. The South African climate (less than 375 mm rain per annum) may be considered harsh, and for cows to survive effectively, some adaptation needs to occur. It is known that *Bos indicus* breed types are more suited to tropical environments, and the *Bos taurus* breed type is more suited to temperate environments [21]. This is an important differentiation to note because of the harsh weather that influenced the conception rate, as it was different between the species that were adapted differently. Weaning affects the development of the calf born to a cow that was weaned via abrupt separation weaning, compared to those using nose clips and late weaning methods [22]. Dairy cows were not milked in this study, but both production types were exposed to weaning processes. The same methods were used for both production types; the only difference was that the synchronization program was suited to the breed type. While being exposed to the same physiological processes, the cows were affected by different climatological factors, showing that adaptation plays a key role in the management of cows in South Africa.

Season affects the comfort zones of cows and is known to influence fertility mainly in dairy cows [23]. High temperatures affect the blood plasma progesterone concentration, oocyte quality and follicular activity [23,24]. Season may influence the number of luteal cells that are produced [8,15]. Most of these studies have only considered dairy cattle. In the current study, there was no effect of the climatological season on the quality of the CL in any synchronization cycles. This could mean factors such as stress may be reduced; there was a full adaptation to the feed and improved body condition (this was not recorded, so we were not able to verify this).

The maximum THI did not affect the beef cows. Beef cows are not known to experience heat stress, but increased body temperature is known to affect reproduction [25]. Ref. [26] showed that beef cattle have a high calving rate up to a THI of 89, and in the current study, only February was above this average, ascertaining that the maximum THI did not affect the beef cows. In the same study, it was shown that a THI above 90 lowered the calving percentage by as much as 30% [26]. The development of the grade of the CL was significantly affected by the daily precipitation that was received by beef cows. This can be linked to low THI readings and cold stress. This production type was more influenced by the THI with the minimum temperature, which can be linked to the adaptation to warmer temperatures for these cows. Cattle are not known to be seasonal breeders [27], but this study shows that they are affected by weather factors.

Heat stress has been shown to alter the colorogenic pituitary hormones, decreasing reproduction in dairy cows [28]. Dairy cattle THI values above 71 should signal the possibility of heat stress [29,30], which is why the THI with the maximum temperature played more of a role in the dairy cow breeds in the current study. An increase in temperature is linked to a decrease in feed intake. This can result in a negative energy balance linked to lower re-conception rates [31].

**Figure 2:**
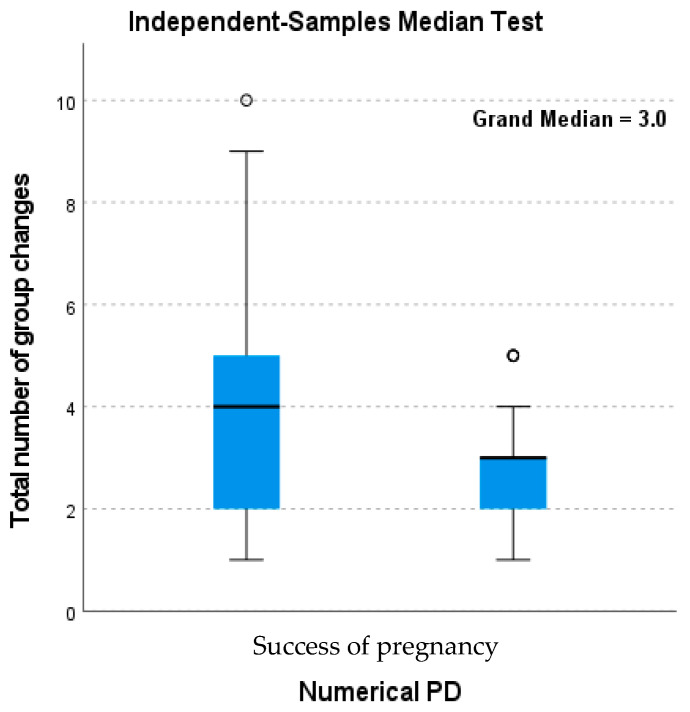
The influence of the number of group changes on the success of pregnancy.

Changing the group of the cow has a strong tendency (*p* = 0.080) to influence the CL quality. Figure 2 shows the success rate of pregnancy within the first cycle. This shows that more group changes in the first cycle resulted in a higher unsuccessful pregnancy rate. Beef cattle are stressed through handling due to the nature of rearing systems [32]. In the current study, the cows were taken off their farm and transported to a facility with a completely new environment and feed. Although they were given a backgrounding period to adapt to the feed, they were exposed to other more constant stressors.

Changing groups means the cows need to obtain new hierarchy and social standings, and that will lead to stress [30]. Lower-ranked cows will produce less Luteinizing Hormone (LH), and this can be linked to the size of the CL. Less LH will lead to lower production of progesterone due to a smaller CL [33].

The synchronization protocol did not influence the quality of the CL; only the first cycle could be considered. The type of program used was matched to the breed type of the specific cow, therefore the program used was best suited for the cow. This can be linked to the CIDR supplying the same progesterone levels to all cows [34]. The lack of a synchronization protocol did not influence the quality of the CL.

## 5. Conclusions

The development of the CL can be improved through a reduction in stressful environments before and during synchronization programs. Different synchronization programs for specific breed types allowed for the synchronization effect to be mitigated. The difference in adaptability between breed types leads to different climate focus areas. In dairy cows, maximum THI, the interaction between average temperature and rain, change in temperature and daily humidity, and the interaction between the change in temperature and wind speed are significant climatological factors. The interaction between the change in temperature and daily humidity was significant in beef cows. More focus needs to be placed on managing the maximum THI and wind barriers for dairy cows, while precipitation and low THI need to be considered for beef cows and for the movement of cows when in calf. Confounding factors need to be considered. Reproduction can be improved through the consideration of climatological and management factors.

## Figures and Tables

**Table 3:** Descriptions of the side, corpus luteum grade and day for luteal characteristics were recorded.

Side	Grade	Day
R—Right Horn	1. Large	1. 8 days after standing estrus
L—Left Horn	2. Medium	2. 7 days after standing estrus
	3. Small	3. 6 days after standing estrus

**Table 4:** Descriptive statistics for climatological variables during the study period (September 2021 to January 2023).

Climatological Factor	n	Minimum	Maximum	Mean	Standard Deviation
Precipitation (mm)	141	0.54	29.47	8.30	18.40
Humidity (%)	483	0.36	10.71	1.79	2.38
Maximum THI (%)	486	58.59	81.14	73.89	5.42
Minimum THI (%)	483	42.55	57.45	50.84	3.73
Wind Speed (km/h)	483	0.04	1.99	0.65	0.51

**Table 5:** The split of beef and dairy breeds for the current study.

Dairy Breeds	Beef Breeds	
CA X JE	AN	CH
HO	AN X	CHX
HO X	BM X	DB
JE	BO	SI
JE X	BO X	SM
	BXX	TU
	BX	NG
	KB X	BR X NG

**Table 6:** Pooled conception rates of recipient cows following the embryo transfer program.

Conception Result	Numerical Value	Percentage
In calf	344	31.1
Not in calf	684	61.84
Unknown	78	7.05

**Table 7:** Effects of anatomical side of ovulation, embryo grade and day of embryo transfer on conception rates of cows and heifers.

Cycle	Factor	n	Test	Value	df	Asymptotic Sig. (2-Sided)
1	Side	520	Pearson Chi-square	0.016	1	0.899
	Grade	520	Linear-by-linear association	2.068	1	0.150
	Day	498	Linear-by-linear association	1.142	1	0.285
2	Side	282	Pearson Chi-square	1.439	1	0.230
	Grade	282	Linear-by-linear association	1.142	1	0.285
	Day	276	Linear-by-linear association	1.809	1	0.179
3	Side	140	Pearson Chi-square	0.146	1	0.703
	Grade	140	Linear-by-linear association	2.607	1	0.106
	Day	138	Linear-by-linear association	0.131	1	0.717

## Data Availability

The original contributions presented in this study are included in the article. Further inquiries can be directed to the corresponding author.

## Abbreviation

The following abbreviation is used in this manuscript:

CL	Corpus Luteum
